# Lung Pathologies and Anatomical Variations in Midwestern American Donor Bodies: Implications for Surgical Planning and Education

**DOI:** 10.7759/cureus.89345

**Published:** 2025-08-04

**Authors:** Sunny Patel, Ilennah Fanega, Peyton Grant, Zainab Ziauddin, Khalid Khan, Umair Naseem, Michael Pollack, Tushar Patel, Shanu Markand

**Affiliations:** 1 Department of Anatomy, A.T. Still University, Kirksville, USA; 2 Department of Pathology, University of Illinois Chicago, Chicago, USA

**Keywords:** lung anatomical variations, lung anatomy, lung pathology, pulmonary fissure grading, thoracic surgical planning

## Abstract

Introduction: Normal anatomical variations between the right and left lungs can affect function and disease presentation; a better understanding of these variations is necessary for optimizing thoracic procedures. Therefore, the current study investigated the lung pathologies of donor bodies to enhance understanding of anatomical variations when performing surgical lung resections, lobectomies, and other thoracic procedures.

Methods: The lungs of 31 donor bodies from A.T. Still University’s Gift of Body Program were analyzed for general pathologies, fissure variations, and tumor characteristics. Specifically, the 62 lungs were examined for pathological changes, completeness of pulmonary fissures, and tumor sizes. To mitigate interobserver bias, eight trained medical students and faculty members collected data using standardized protocols to ensure consistency in fissure grading and pathology identification.

Results: Pulmonary fibrosis and atelectasis were the most frequent pathologies, both of which were observed in 11 (17.7%) lungs. Lung cancer was found in eight lungs (12.9%), right heart enlargement in four (6.6%), pulmonary emphysema in four (6.6%), and bronchopneumonia in four (6.6%). Of 62 fissures, 15 (24.2%) were separable at the hilum. Although not statistically significant, a trend was observed in which lower fissure grades were associated with larger average tumor sizes. Specifically, tumors in Grade 1 lungs averaged 134.93 mm compared to 95.67 mm in Grade 2 and 108.43 mm in Grade 3 fissures. The mean (SD) diameter of tumors was 111.58 (38.48) mm. No difference in tumor diameter was found between the left and right lungs.

Conclusion: Overall, study results indicated a correlation between higher fissure grades and severe pathologies, particularly in cases with Grade 3 fissures. These findings may provide valuable insights for optimizing surgical planning in lung resections, lobectomies, and related procedures.

## Introduction

The lungs are paired organs positioned laterally to the mediastinum, just above the diaphragm, and are composed of highly branched bronchial structures and alveoli, which are essential for respiration [1]. Although the right and left lungs are functionally similar, the right lung is broader and shorter to accommodate the liver’s position, and it contains three lobes separated by horizontal and oblique fissures [1]. In contrast, the left lung is smaller and narrower, with a slight depression to accommodate the heart and two lobes separated by an oblique fissure [2]. These anatomical variations influence normal function and disease presentation, making them crucial for understanding lung pathology.

Anatomical variations often originate during early lung development [3]. Embryogenesis is a highly regulated process where even minor structural deviations can lead to long-term functional consequences [3]. During the fourth week of gestation, the ventral foregut endoderm gives rise to the respiratory epithelium, forming the trachea and bronchial tree [4]. Lung development then progresses through five stages: embryonic, pseudoglandular, canalicular, saccular, and alveolar [5]. The lungs undergo extensive differentiation throughout these stages, making them highly susceptible to anatomical variations that may impact function later in life [6]. As noted in previous reviews [3], the right lung’s horizontal fissure is less prevalent than oblique fissures, and incomplete or absent horizontal fissures are more common. Because anatomical differences can influence disease presentation and surgical outcomes, a better understanding of possible lung variations is crucial, highlighting the need for additional research on fissure development.

From a surgical perspective, knowledge of possible anatomic variations is vital, as lung resections and lobectomies are often planned using natural anatomic landmarks [7-11]. However, the lungs are highly adaptable organs, and their anatomical structure and physiological function are constantly shifting in response to extrinsic and intrinsic factors, such as diseases, tumors, and aging [12]. Thus, assessing anatomical patterns is crucial for improving intraoperative navigation during these procedures [13]. As reported by Li and Che [14] and Li et al. [15], poorly developed pulmonary fissures strongly predicted postoperative complications after video-assisted thoracic surgery. Li and Che [14] and Li et al. [15] also noted an increase in cardiopulmonary complications associated with poorly developed fissures and recommended integrating pulmonary fissure completeness assessments into surgical training programs.

To address anatomical variations that complicate standard tumor resection surgeries, researchers developed the adaptive magnetic resonance-guided stereotactic radiotherapy technique [16]. However, examination of how different pathologies alter lung structure is still necessary to better understand anatomical changes for the successful planning of interventional procedures. Current efforts to address anatomical variation, such as adaptive MRI-guided stereotactic radiotherapy [16], have improved surgical precision; however, continued research remains necessary to support interventional procedure planning. Additionally, while embryological development is well characterized, few cadaveric studies have analyzed anatomical and pathological variations, particularly fissure completeness, in donor lungs.

The primary objective of this study was to investigate general lung pathologies, the completeness of pulmonary fissures, and tumor characteristics in human donor lungs. We hypothesized that a higher fissure grade would be correlated with larger tumor sizes. Our goal was to improve understanding of anatomical variation in pathology, to enhance surgical planning, preoperative assessment, and medical education. These findings may also inform radiologic interpretation, imaging, and interventional strategies during lung resections and lobectomies.

## Materials and methods

All procedures in the current study were considered exempt by the A.T. Still University-Kirksville Institutional Review Board. The study was conducted at A.T. Still University’s Kirksville College of Osteopathic Medicine. It included 31 donor bodies, comprising 20 men and 12 women aged 34-98 years, obtained through A.T. Still University’s Gift of Body Program.

After thoracic cavity dissection, both lungs were isolated and weighed using an Ohaus Adventurer™ Precision Balance (model AX2202, Ohaus Corporation, Parsippany, NJ; capacity: 2,200 g, readability: 0.01 g). One pair of lungs was excluded from the study due to damaged tissue. The remaining lungs were then examined for structural variations, pathological findings, and tumor pathology before being photographed with an iPhone 14 Pro Max (Apple Inc., Cupertino, CA; 48 MP main camera) under consistent lighting to ensure high-detail documentation.

Outcome measures

General Pathologies

Each lung was closely inspected for common pathological conditions, including pulmonary fibrosis, atelectasis, lung cancer, and other structural abnormalities. The findings were documented, and the donor body’s cause of death was recorded.

Pulmonary Fissure Completeness

The extent of pulmonary fissure completeness was evaluated through visual inspection and palpation to identify complete or incomplete separations between lung lobes. To reduce interobserver bias and to ensure consistent and accurate assessments, data were analyzed in a blinded manner by eight medical students, an anatomy faculty member, and a pathologist. A modified grading scale based on Kaur et al. [17] was adapted to assess completeness. Grade 0 indicated the fissure was absent. Grade 1 meant that an external marking of the fissure was present, but the lobes were not separable. Grade 2 indicated the fissure was partially developed and could be separated to some extent. Grade 3 indicated a fully formed fissure that allowed complete separation of the lobes at the hilum.

Tumor Presence and Size

If a tumor was present, its diameter was measured using ImageJ (National Institutes of Health, Bethesda, MD, USA), and its location and characteristics were documented.

Statistical analysis

Data were analyzed using Microsoft Excel (Microsoft Corporation, Redmond, WA). Lung pathology outcomes were summarized using frequency and percentage. A two-sample t-test was used to determine whether there were differences in lung pathology between the left and right lungs. A Pearson correlation coefficient was used to assess relationships between tumor size, pathological findings, and fissure grades. A one-way analysis of variance was used to test for differences in tumor sizes by fissure grades. A p value of <0.05 was considered statistically significant.

## Results

General pathologies

**Figure 1:**
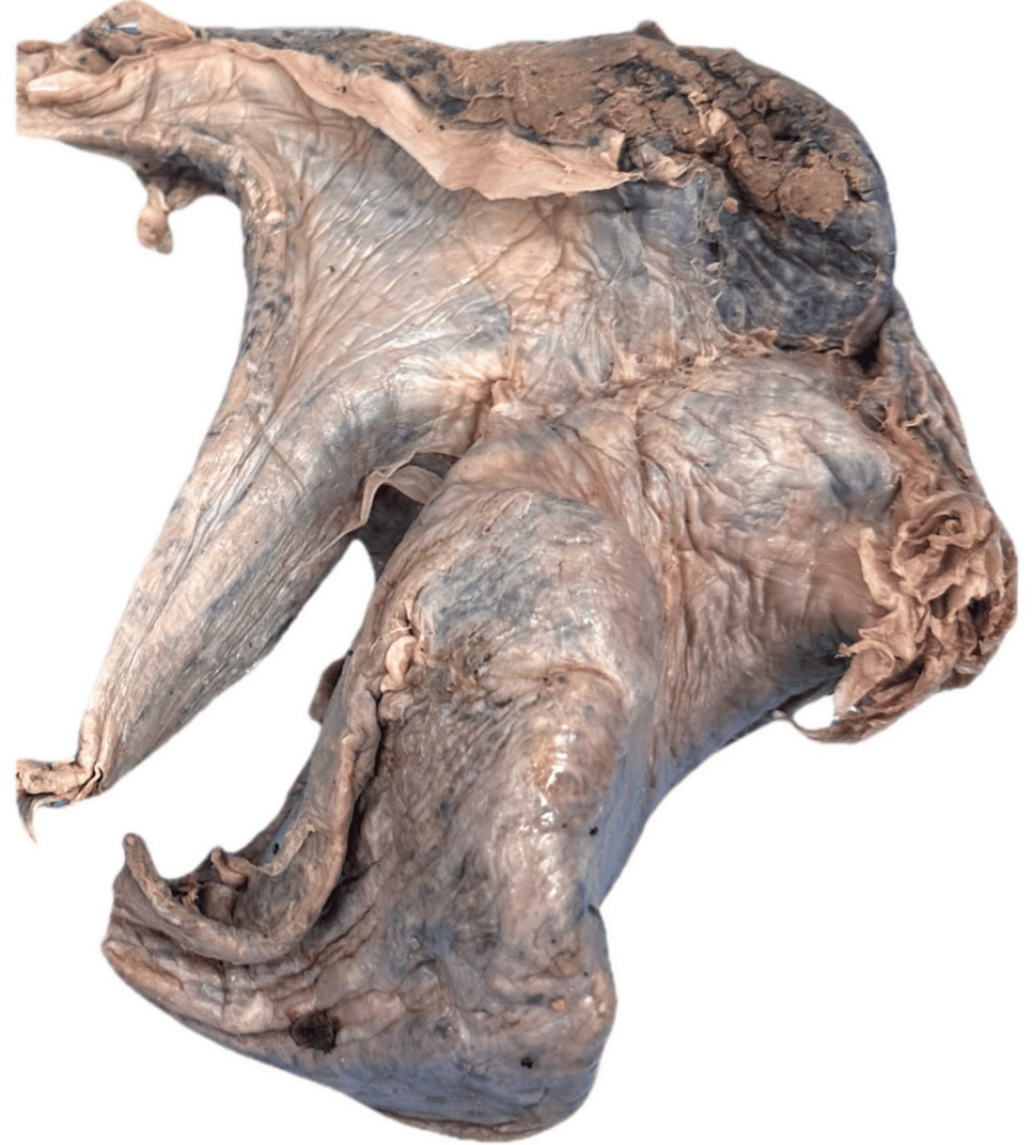
Atelectasis The image demonstrates a markedly shrunken and collapsed lung with volume loss, consistent with atelectasis. The wrinkled and concave surface reflects loss of air from the alveoli and reduced inflation

**Figure 2:**
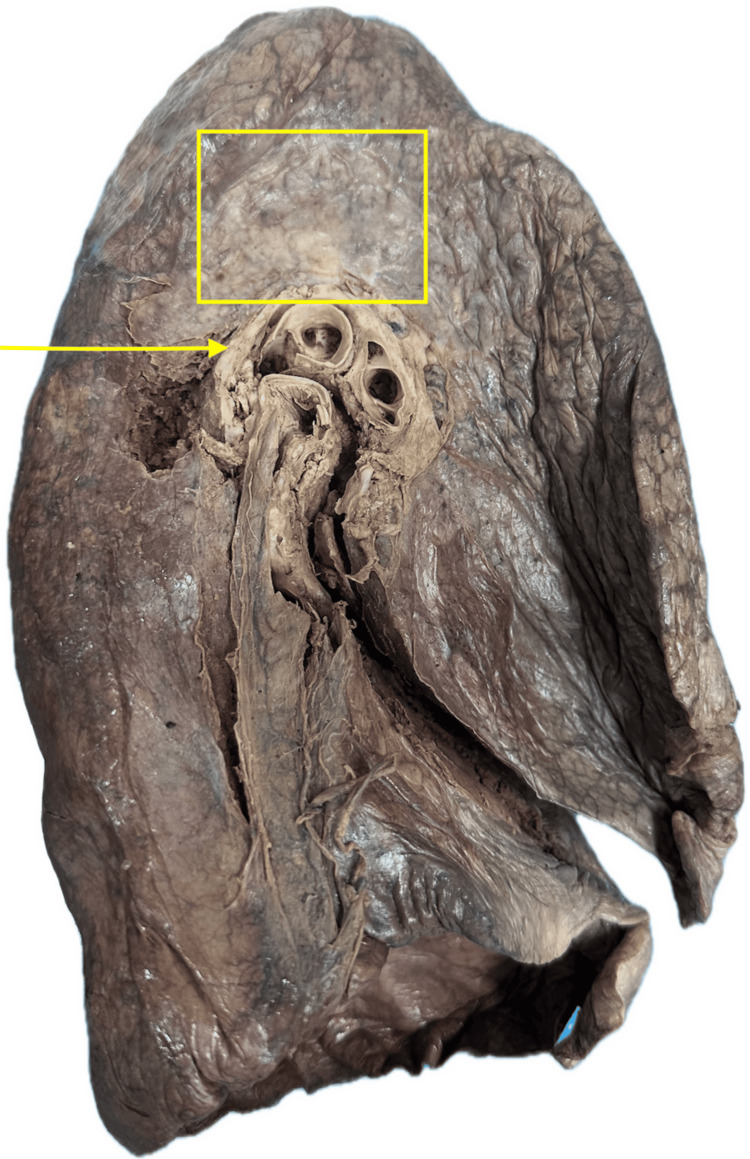
Pulmonary and parenchymal fibrosis The arrow indicates bronchial distortion and thickening, suggestive of airway remodeling. The boxed area highlights dense fibrotic parenchyma with architectural distortion. Lungs with pulmonary and parenchymal fibrosis had stiffened tissue, retraction of fissures, and a leathery fibrotic structure

Of the 62 donor lungs examined, atelectasis (Figure 1) and pulmonary fibrosis (Figure 2) were the most prevalent conditions, each identified in 11 specimens (17.7%).

**Figure 3:**
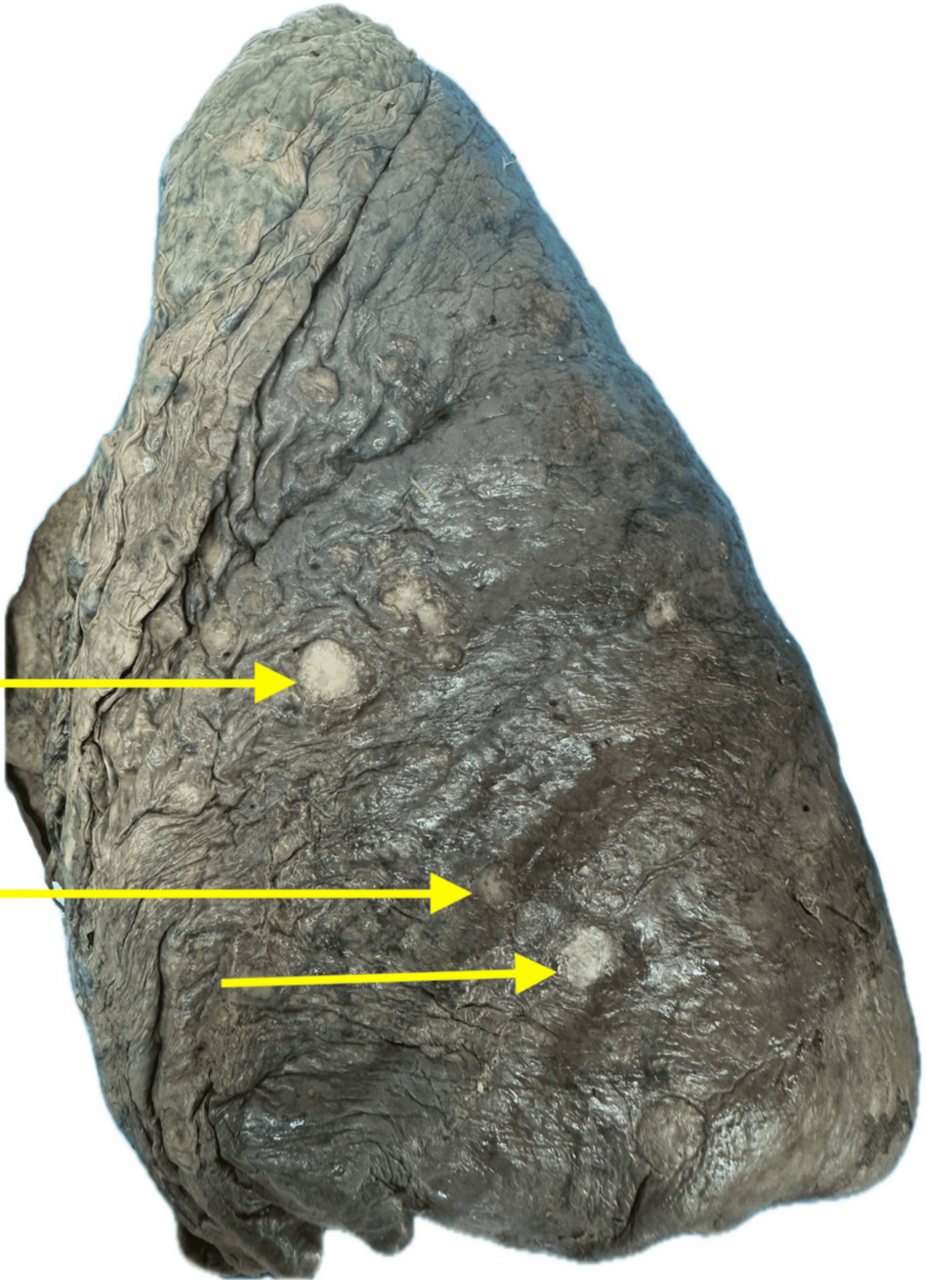
Lung with tumorigenic changes The irregular, demarcated borders observed suggested tumorigenic changes. The arrows indicate distinct areas of pleural distortion and surface nodularity, consistent with underlying tumor pathology

**Figure 4:**
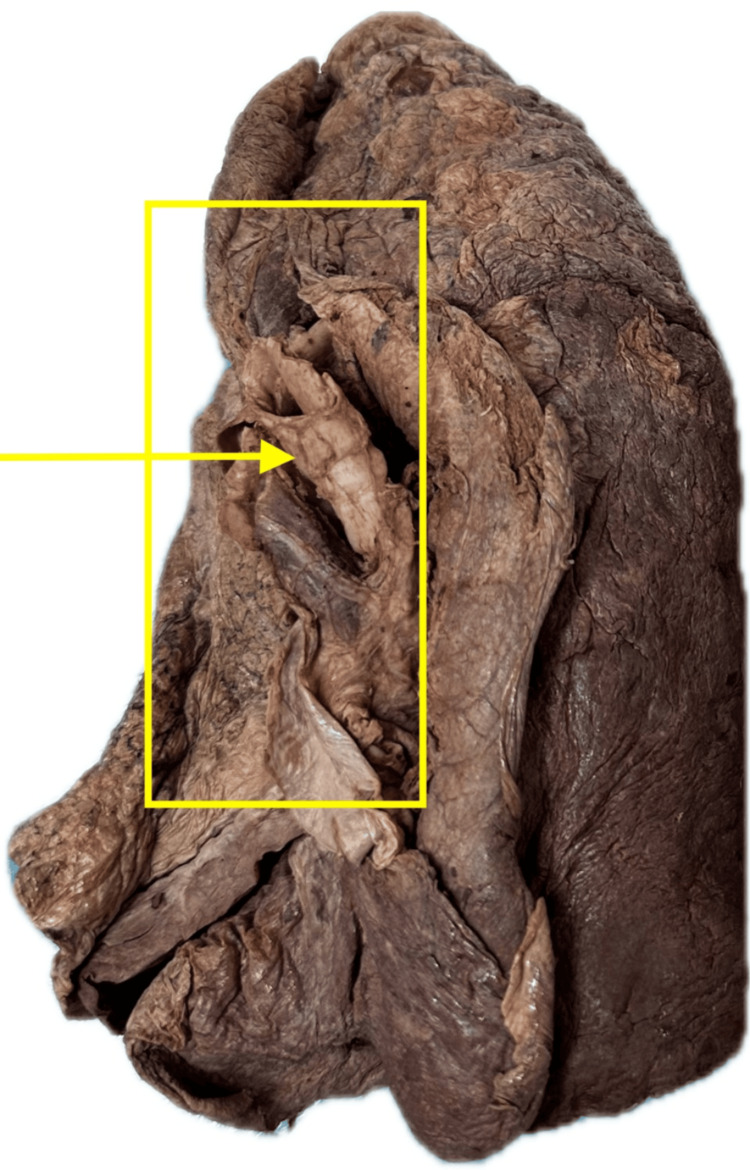
Lung affected by right heart enlargement The arrow indicates vascular congestion and compression near the hilum, while the boxed areas show fibrotic changes and narrowing of pulmonary vasculature from chronic right-sided heart strain

Lung cancer (Figure 3) was observed in eight specimens (12.9%), although no laboratory confirmation was performed to verify the diagnosis. Other findings included damage associated with right heart enlargement (Figure 4) in four specimens (6.5%), pulmonary emphysema in four specimens (6.5%), and bronchopneumonia in four specimens (6.5%). No pathological abnormalities were found in 20 lungs (32.3%).

Fissure grades

Of the 62 lungs, 10 (16.1%) were classified as Grade 1, 37 (59.7%) as Grade 2, and 15 (24.2%) as Grade 3. No lungs were classified as Grade 0.

Tumor size

Of 62 donor lungs, 12 (19.4%) had findings consistent with potential lung tumors. The mean (SD) tumor diameter was 111.58 (38.48) mm. Left lung tumors had a mean (SD) diameter of 111.37 (31.90) mm, and the right lung tumors had a mean (SD) diameter of 111.76 (47.85) mm. When tumor sizes between the left and right lungs were compared, no difference was found (p = 0.99).

Comparisons of study outcomes

When evaluating the relationships between tumor size, pathological findings, and fissure grades, a moderate correlation (r = 0.64) was found between tumor size and pathological findings. The mean tumor size by fissure grade is presented in Table 1. When comparing these outcomes, a weak positive correlation (r = 0.32) was found, but no difference was found in tumor sizes by fissure grades (p = 0.55).

**Table 1:** Mean tumor size by fissure grade (n = 62)

Fissure grade	Tumor size, mm, mean (SD)
Grade 0 (n = 0)	Not applicable
Grade 1 (n = 10)	134.93 (17.18)
Grade 2 (n = 37)	95.67 (18.76)
Grade 3 (n = 15)	108.43 (41.99)

## Discussion

Lung development and anatomical variability

The current study evaluated the lung pathologies of 31 donor bodies from the Midwestern United States. Analysis of general lung pathologies, pulmonary fissure completeness, and tumor characteristics indicated a correlation between higher fissure grades and severe pathologies, particularly in cases with Grade 3 fissures. Because pathological changes can have a major impact on lung structure and function, these results contribute to the literature on anatomical variations. They may provide valuable insights for clinical practice and pulmonary research. These findings may offer preliminary insights for clinical practice and pulmonary research but should be interpreted cautiously given the limited sample size.

A better understanding of lung pathologies is crucial for enhancing the comprehension of anatomical variations when performing surgical lung resections, lobectomies, and other thoracic procedures. For example, improved information about disease prevalence may be useful for identifying common pulmonary conditions and reinforcing patterns observed in clinical settings. Furthermore, a deeper understanding of fissure variations may offer insight into lung development during embryogenesis. During surgical planning, knowledge of the prevalent types and grades of fissure variations may be vital for successful procedures. Similarly, the ability to recognize lung tumors may improve the identification of the potential patterns that contribute to disease progression and the anatomical factors that influence tumor growth.

The current study's results on fissure variations were supported by previous research [14,15,18,19]. For example, West et al. [18] also reported considerable variability in pulmonary fissure completeness across populations. However, when comparing fissure completeness and tumor size, we found a weaker correlation than that reported in other studies [8,14,15]. Research has also shown evidence of tumors crossing fissures of varying completeness [14,15,19]. Overall, these findings suggest that factors other than fissure completeness contribute to the development and progression of lung tumors. More research is necessary for a better understanding of lung pathologies and their effect on surgical outcomes.

A first step in improving the understanding of fissure variations involves considering lung development during embryogenesis. Beginning in the fourth week of gestation, the lungs undergo a series of five stages of embryological development [5]. At that point, any incomplete separation can lead to poor fissure formation. As the lungs continue to develop and expand, this potential pathology may be accelerated or decelerated later in life [20]. Many disease processes, such as emphysema, have been linked to embryological development and its role in lung formation [20]. The results of the current study support the connection between lung development and lung pathology, reinforcing the effects of early structural differences on variations in disease presentation and progression.

In addition to embryological development, structural changes that occur over time due to chronic inflammation, lung injury, or tissue remodeling are also major factors in lung pathology [21]. These changes may arise from prolonged exposure to irritants [21], insufficient lung expansion during prolonged mechanical ventilation, or conditions related to immobility [22]. Additionally, alterations in lung fissures and overall architecture may contribute to disease progression [23], thereby impacting disease development, ventilation dynamics, and surgical considerations. In normal lung anatomy, the right lung has two primary fissures and the left lung has only one. However, we found no statistically significant difference for tumor presence or distribution. This finding suggested that tumor spread is more likely influenced by other factors, such as underlying primary malignancy and vascular or lymphatic drainage, than by fissure anatomy [24]. Therefore, an understanding of these pathophysiological changes may lead to a better characterization of lung disease development and progression, as well as improved diagnostic accuracy and treatment strategies.

Given the importance of fissure variations and their effect on lung pathologies, more research investigating pulmonary disease progression is necessary. From a surgical perspective, incomplete fissures can complicate lung resection surgeries by increasing the risk of prolonged air leakage and hospital stays [25], particularly during minimally invasive procedures involving video-assisted thoracic surgery [14,15] or robotic-assisted thoracic surgery [26]. Because anatomical landmarks are crucial during surgical interventions, the absence or incompleteness of these landmarks may lead to additional tissue dissection, thereby increasing operative time and potentially resulting in postoperative complications, such as pulmonary edema, pneumonia, and pleural effusion [14,15]. Similarly, the presence of lung tumors with complete or incomplete fissures may influence surgical outcomes, but this relationship remains speculative and warrants further investigation in larger sample sizes. For example, if incomplete fissures allow for larger tumor distribution, surgical planning and radiation therapy protocols will likely need to account for these anatomical differences. Because lung pathology varies among individuals, recognition of these changes is essential for targeted treatment and surgical planning approaches. By personalizing clinical management to account for anatomical differences, patient outcomes can be optimized.

Limitations

A significant limitation of the current study was the relatively small sample size, which may affect the generalizability of the study findings. Additionally, the donor bodies included in the study were from a specific region of the United States, which may have limited the robustness of our statistical conclusions due to this homogeneity. Evaluation of lungs relied on gross morphology, and histological confirmation of findings was not performed. This lack of confirmation led to misclassification of certain lungs, potentially overestimating the number of lungs with tumor pathology. Additionally, confounders such as age, occupational exposure, smoking status, and comorbidities were not available or controlled for, which may have influenced the distribution of pathologies alongside interobserver variability. Although our results suggested a moderately weak correlation between tumor presence and fissure completeness, additional studies involving larger sample sizes are necessary.

Future directions

To enhance understanding of anatomical variations during thoracic procedures, future studies should investigate the role of fissure completeness in metastatic and de novo lung cancers, assessing whether variations in fissure structure influence tumor progression and spread. Additionally, evaluating potential correlations between fissure completeness, survival outcomes, recurrence rates, and lymphatic involvement may yield clinically meaningful insights that optimize patient care and treatment strategies.

## Conclusions

The results of the current study highlighted common lung pathologies and anatomical variations in Midwestern American donor bodies, suggesting potential correlations between general lung pathologies, fissure completeness, and tumor characteristics. It emphasized the importance of considering structural variability during the surgical planning process. Ultimately, a better understanding of potential differences in lung structure and their relationship to disease progression may lead to more refined treatment approaches and improved patient outcomes. Such information would also be invaluable for guiding future research in pulmonary medicine.
